# Assessment of maternal and child health care services performance in the context of COVID-19 pandemic in Addis Ababa, Ethiopia: evidence from routine service data

**DOI:** 10.1186/s12978-022-01353-6

**Published:** 2022-02-14

**Authors:** Senedu Bekele Gebreegziabher, Solomon Sisay Marrye, Tsegaye Hailu Kumssa, Kassa Haile Merga, Alemu Kibret Feleke, Degu Jerene Dare, Inger Kristensson Hallström, Solomon Abebe Yimer, Mulatu Biru Shargie

**Affiliations:** 1 Armauer Hansen Research Institute (AHRI), Addis Ababa, Ethiopia; 2 KNCV, Addis Ababa, Ethiopia; 3 Yekatit-12 Hospital Medical Collage Public Health Department, Addis Ababa, Ethiopia; 4 KNCV Tuberculosis Foundation, Hague, The Netherlands; 5 Department of Health Sciences, Child and Family Health, Lund University, Stockholm, Sweden; 6 Vaccine Research and Development Department, Coalition for Epidemic Preparedness Innovations (CEPI), Oslo, Norway; 7 Faculty of Medicine, Unit for Genome Dynamics, University of Oslo, Oslo, Norway

**Keywords:** COVID-19, Pandemic, Maternal, Child, Health services, Routine service data, Addis Ababa, Ethiopia

## Abstract

**Background:**

In many settings, health care service provision has been modified to managing COVID-19 cases, and this has been affecting the provision of maternal and child health services. The aim of this study was to assess trends in selected maternal and child health services performance in the context of COVID-19 pandemic.

**Methods:**

A cross-sectional data review was conducted in Addis Ababa, Ethiopia from April to May 2021. Routine health management information system database was reviewed from Addis Ababa Health Bureau for the period from July 2019 to March 2021 across all quarters. Proportion and mean with standard deviation were computed. T-test was used to assess statistically significant differences in services mean performance.

**Results:**

Postnatal care  visit, new contraceptives accepters, safe abortion care and number of under-5 years old children treated for pneumonia significantly decreased by 9.3% (p-value 0.04), 20.3% (p-value 0.004), 23.7% (p-value 0.01) and 77.2% (p-value < 0.001), respectively during the first 8 months of the COVID-19 pandemic compared to the previous 8 months’ average performance. The trends in Antenatal care first visit, new contraceptive accepters, pentavalent-3 vaccination and under-five children treated for pneumonia began to decline in January to  March 2020, a quarter when the COVID-19 pandemic began; with accelerated declines in April to June 2020 following national lockdown. The trends for the stated services began to increase during July–September 2020, the last quarter of national lockdown. Contraceptive accepters and pentavalent-1 vaccination continued to decline and showed no recovery until January–March 2021 when this study was completed.

**Conclusions:**

Most of the maternal and child health services performance declined following the onset of COVID-19 pandemic and national lockdown, and most of the services began recovering during July–September 2020, the last quarter of national lockdown. However, new and repeat contraceptive accepters and pentavalent-1 recipients continue to decline and show no recovery during end of the study period. Implementing COVID-19 prevention measures and assuring the community about the safety of service delivery is imperative to ensure continuity of the maternal and child health services. Regular monitoring and evaluation of services performance is required to identify slowly recovering services and respond to potentially volatile changes during the COVID-19 pandemic.

## Plain English summary

The coronavirus disease 2019 (COVID-19) is the worst global health crisis of a century. In many settings, health care service provision has been modified to focus on managing COVID-19 cases, and this has been affecting the provision of health services including maternal and child health services. The aim of this study was to assess the trends in maternal and child health services performance in the era of COVID-19 pandemic in Addis Ababa, Ethiopia. The results of the studies showed post-delivery care, new family planning attendees, safe abortion care and number of under-5 years old children treated for pneumonia decreased during the first 8 months of the COVID-19 pandemic compared to the previous 8 months’ average performance. The trends in antenatal care (care during pregnancy), new family planning attendees, pentavalent-3 vaccination and under-five children treated for pneumonia began to decline between January-March 2020, a quarter when the COVID-19 pandemic began; with accelerated declines in April to June 2020 following national lockdown. The trends for the stated services began to increase during July–September 2020, the last quarter of national lockdown. Family planning attendees and pentavalent-1 vaccination continued to decline and showed no recovery until January–March 2021when this study was completed. Implementing COVID-19 prevention measures and assuring the community about the safety of service delivery is imperative to ensure continuity of the maternal and child health services. Regular monitoring and evaluation of services performance is required to identify slowly recovering services and respond to potentially volatile changes during the COVID-19 pandemic.

## Background

The coronavirus disease 2019 (COVID-19) pandemic is the worst global health crisis of a century declared as a Public Health Emergency of International Concern by the World Health Organization (WHO) in March 2020 [1]. As of October 22, 2021, more than 243 million confirmed COVID-19 cases and 4,945,928 related deaths have been reported globally. The Africa region reported 8,528,131 cases and 216,956 related deaths in the same period [2].

The COVID-19 pandemic has challenged the resilience of the most effective health systems in the world [1]. In many settings, health care service provision has been modified to focus on managing COVID-19 cases, and this pandemic focused approach has been affecting the provision of routine health services including reproductive, maternal, neonatal and child health (RMNCH) services [3]. Essential health services including antenatal care (ANC), skilled birth attendance (SBA), postnatal care (PNC), and other sexual and reproductive health care services such as family planning (FP), post-abortion care, and, where legal, safe abortion services to the full extent of the law need to remain available [4]. The WHO recommends continuation of essential health services including maternal and child health (MCH) services regardless of COVID-19 expansion taking into consideration the implication of interruption of those services in health care facilities [5]. However, many countries, particularly countries in Africa have faced substantial challenges to maintain the provision of high quality essential RMNCH services during COVID-19 pandemic [6]. The health care system in Africa faces several challenges such as: lack of funds, inequitable allocation of resources to the health sector, low healthcare workforce, high disease burden and inadequate health infrastructure. The already fragile health system in most countries in Africa is overburdened with additional mission to address COVID-19 pandemic [7].

Ethiopia reported the first confirmed COVID-19 case on 13th March 2020 [8]. Consequently, the government declared a national state of emergency on 8th April 2020 that would last until 10th September 2020 [9]. The country adopted a variety of response measures to mitigate the infection and halt the spread of the severe acute respiratory syndrome coronavirus 2 (SARS-CoV-2) [10]. The measures included travel restrictions, suspension of international flights, international border closure, mandatory quarantines, flexible working arrangements, suspending public gathering and sports, closure of schools and universities, requirements for social distancing and others [10, 11]. Despite all the measures taken, however, the country reported the highest number of confirmed COVID-19 cases in the East Africa region. As of October 22, 2021, Ethiopia reported 361,027 confirmed cases and 6,316 COVID-19 related deaths [2]. Addis Ababa, the capital city of Ethiopia comprised more than 50% of total confirmed COVID-19 cases within the country [12].

The government of Ethiopia has identified RMNCH services as essential health services for improving maternal, neonatal and child health [13]. Substantial achievements in reducing maternal mortality ratio, under-5 and infant mortality rates have been documented during the last two decades in the country by implementing essential health services [14]. Maintaining access and utilization of essential health services in the era of the COVID-19 pandemic is crucial to prevent unfavorable outcomes and protect the gains made over the past years in reducing maternal, infant and child mortality rates [15]. In April 2020, the Ministry of Health-Ethiopia set a guide for maintaining essential health services during the COVID-19 pandemic [16].

Several studies from different countries around the world showed the negative impacts of COVID-19 pandemic on MCH services [17–21]. In Ethiopia, some studies from various regions showed reductions in MCH services during the early period of the COVID-19 pandemic [6, 11, 22–24]. However, trends in the MCH care services performance during and after national lockdown have not been adequately assessed, particularly in Addis Ababa, the capital city where most COVID-19 cases were detected. The aim of this study was to assess trends in selected RMNCH services performance indicators before, during and after national lockdown in the context of COVID-19 pandemic in Addis Ababa, Ethiopia.

## Methods

### Study design and setting

A cross-sectional data review was conducted in Addis Ababa City Administration Health Bureau (AACAHB), Ethiopia from April to May 2021 to assess trends in selected MCH health services performance in the context of COVID-19 pandemic. Addis Ababa is the capital city of Ethiopia, and is one of the most densely populated cities in the country, with an estimated population of more than five million in 2021 [25]. Ninety-five government health centres, 56 public hospitals and 66 private health care facilities are providing health services including MCH services in the city [26]. AACAHB is responsible for the health-care administration in the city.

### Data sources and management

Routine health management information system (HMIS) RMNCH services database from AACAHB was reviewed to assess trends of selected MCH services performance indicators for the period starting from July 2019 to March 2021 across all quarters. The period from July 2019 to March 2021 covers the periods before, during and after lockdown time periods in Ethiopia. Two trained data collectors reviewed the data from April to May 2021. Data collectors were trained about the entire process of data collection including quality control measures such as: completeness, correctness, consistency, and synchronizing and archiving data with RedCap. The data reviewed included ANC, births attended by skilled health personnel at a health facility, early PNC (within seven days after delivery), contraceptives accepters, safe abortion care (abortion care provided at the time of induced abortion), post abortion care (abortion care provided during spontaneous abortion), early neonatal deaths (institutional neonatal deaths in the first 7 days of life), under-five children pneumonia treatment and immunization services. Regular supervision and follow-up were made throughout the data collection period. Completeness, correctness and consistency of the reviewed data were checked daily by supervisors. The overall activities and entire process of data collection were led by the principal investigator.

Precaution measures including maintaining wearing face mask, using hand sanitizers and physical distancing were implemented to prevent COVID-19 transmission during data collection.

### Data analysis

The reviewed routine HMIS data were cleaned, checked for consistency and entered into the Redcap database, and the cleaned data were exported into Stata version15 statistical software package for statistical analysis. Descriptive statistics such as proportion and mean with standard deviation (SD) were computed. To assess changes in RMNCH services during the early period of COVID-19 pandemic, we compared the average services performance for the period March to October 2020 (the first 8 months of COVID-19 transmission in Ethiopia) with the July 2019 to February 2020 period (the preceding 8 months before COVID-19 transmission started). To assess trends in MCH services performance in the pre-COVID period, during and after lockdowns, trends of selected RMNCH health services indicators were evaluated across quarters from July 2019 to March 2021. We used the first 3 months (July–September 2019) mean performance as a baseline to compute the relative percentage changes of the services performances for each quarter. T-test was computed to assess statistically significant differences in services mean performance. Test of normality was checked by histogram. A p-value of < 0.05 was considered statistically significant.

### Ethical approval

The Armauer Hansen Research Institute (AHRI) ethics review committee and the Addis Ababa City Administration ethics review committee approved this study. In addition, permission to review routine HMIS data was obtained from AACAHB authority.

## Results

### Maternal and child health services performance during the first 8 months of COVID-19 pandemic period

Early PNC visit, new contraceptives accepters and safe abortion care significantly decreased by 9.3% (p-value 0.04), 20.3% (p-value 0.004) and 23.7% (p-value 0.01), respectively during the first 8 months (March–October 2020) of the COVID-19 pandemic compared to the previous 8 months’ average performance. Similarly, there was a decrease in Pentavalent-1 vaccination (0.3%), Pentavalent-3 vaccination (4.7%) and fully vaccination (0.6%) in the same period after COVID-19 pandemic. Measles first dose vaccination increased by 1.7% during March–October 2020 compared to the period July 2019–February 2020 average performance. The average number of under-5 years old children treated for pneumonia was significantly reduced by 77.2% (p-value < 0.001) in the period March–October 2020. However, there were increment in ANC four or more visits (+ 1.2%) and post abortion care (+ 17.8%) during the same period (Table 1).

**Table 1:** Maternal and child health services during the first 8 months of COVID-19, Addis Ababa, Ethiopia

Health services	Indicators	July 2019–February 2020 Baseline mean	March–October 2020 Mean	Percentage change (%)	p-value
Maternal health services	ANC first visit	13,780.75	12,811	− 7.0	0.15
	ANC four or more visits	10,469.75	10,597.75	+ 1.2	0.79
	Health facility births	11,745.13	11,454.13	− 2.5	0.50
	Early PNC visit	11,588.13	10,514.5	− 9.3	0.04*
	New contraceptives accepters	10,054.5	8014.5	− 20.3	0.004*
	Repeat contraceptives accepters	18,742.5	16,967.38	− 9.5	0.10
	Safe abortion care	2739.5	2091.25	− 23.7	0.01*
	Post abortion care	1010.5	1189.87	+ 17.8	0.21
Child health services	Pentavalent-1 vaccination^a^	11,037.88	11,008.88	− 0.3	0.94
	Pentavalent-3 vaccination^b^	11,198.5	10,667.25	− 4.7	0.27
	First dose measles vaccination	9990.37	10,160.5	1.7	0.86
	Fully vaccination	9626.37	9572.87	− 0.6	0.95
	Under five children pneumonia treatment	4861.87	1108.37	− 77.2	< 0.001*

This table shows the maternal and child health services performance during the first 8 months (March–October 2020) of the COVID-19 pandemic period compared to the baseline (July 2019–February 2020) average performance, Addis Ababa, Ethiopia

^a^First dose pentavalent vaccination

^b^Third dose pentavalent vaccination

*p-value < 0.05

The average number of institutional neonatal deaths in the first 7 days of life in the periods covering July 2019 to February 2020 and March to October 2020 was 105 and 119, respectively. Institutional neonatal deaths increased by 13.3% from July 2019 to February 2020 to March to October 2020 (p-value = 0.02).

### Trends in the maternal and child health services

**Fig. 1:**
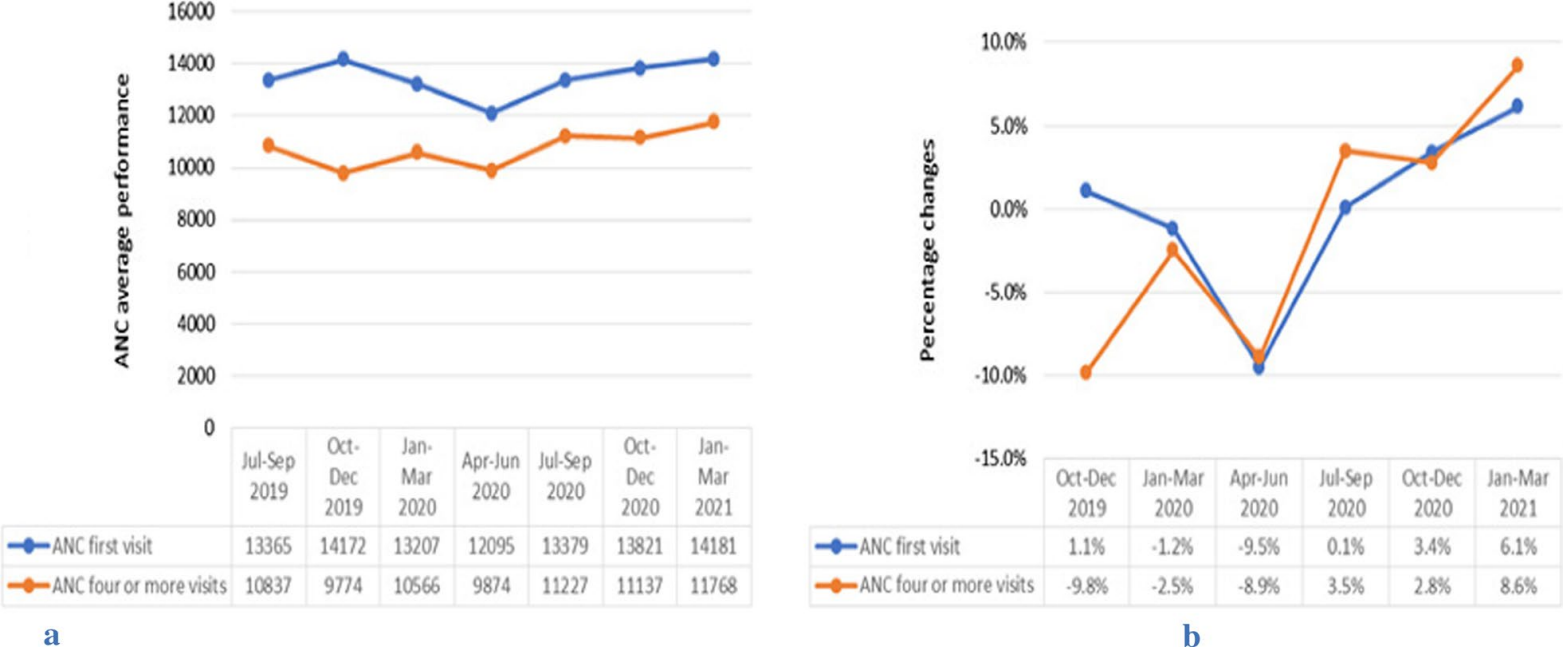
Trends in mean number and percentage changes in ANC visits across quarters.  **a** shows trends in mean number of ANC visits across quarters from July 2019 to March 2021 and **b** shows percentage changes in ANC visits across quarters from October 2019 to March 2021 compared to baseline (July–September 2019) average performance, Addis Ababa, Ethiopia

Services trend analysis across quarters for the period July 2019 to March 2021 showed that ANC first visit began to decrease in the period January to March 2020 (a quarter at the onset of COVID-19 in Ethiopia), with accelerated decline during April–June 2020 (national lockdown declared to control COVID-19). ANC four or more visits began declining during October–December 2019 (before onset of COVID-19 pandemic in Ethiopia), and began to recover in January to March 2020 then declined in the period April–June 2020 (Fig. 1a). Compared to the baseline performances, quarterly percentage changes in ANC visits across quarters for the period October 2020 to March 2021 showed that ANC first visit reduced by 9.5% for the period April–June 2020 compared to the July–September 2019 average performance. ANC four or more visits reduced by 9.8% and 8.9%, respectively during October–December 2019 and April–June 2020 compared to the July–September 2019 average performance (Fig. 1b). ANC first and four or more visits started to increase during the period July–September 2020 (the second quarter of national lock down) to a level higher than pre-pandemic baseline level (Fig. 1a).

Early PNC visit slightly declined in the periods October–December 2019 and January–March 2020, with accelerated decline during April–June 2020. For the period April–June 2020, early PNC visit decreased by 17.5% compared to the July–September 2019 average performance. PNC visit continuously increased across quarters in the periods July to September 2020, October to December 2020 and January to March 2021. The percentage change in early PNC visit across quarters of the study periods was statistically significant with p-values 0.04 and 0.05 in April–June 2020 and July–September 2020, respectively (Table 2).

**Table 2:** Postnatal care across quarters for the period July 2019–March 2021, Addis Ababa, Ethiopia

Months/quarters	Early PNC visit Frequency (N)	Early PNC visit (mean ± SD)	Percentage change	p-value
July–September 2019	35,233	11,744.33	Baseline	–
October–December 2019	34,323	11,441±1936.52	− 2.6%	0.81
January–March 2020	34,098	11,366 ± 1292.48	− 3.2%	0.67
April–June 2020	29,042	9680.66 ± 717.86	− 17.5%	0.04*
July–September 2020	32,344	10,781.33 ± 393.84	− 8.2%	0.05*
October–December 2020	33,031	11,010.33 ± 1899.69	− 6.3%	0.57
January–March 2021	35,135	11,712 ± 1341.91	− 0.3%	0.97

*SD* standard deviation

*p-values < 0.05

**Fig. 2:**
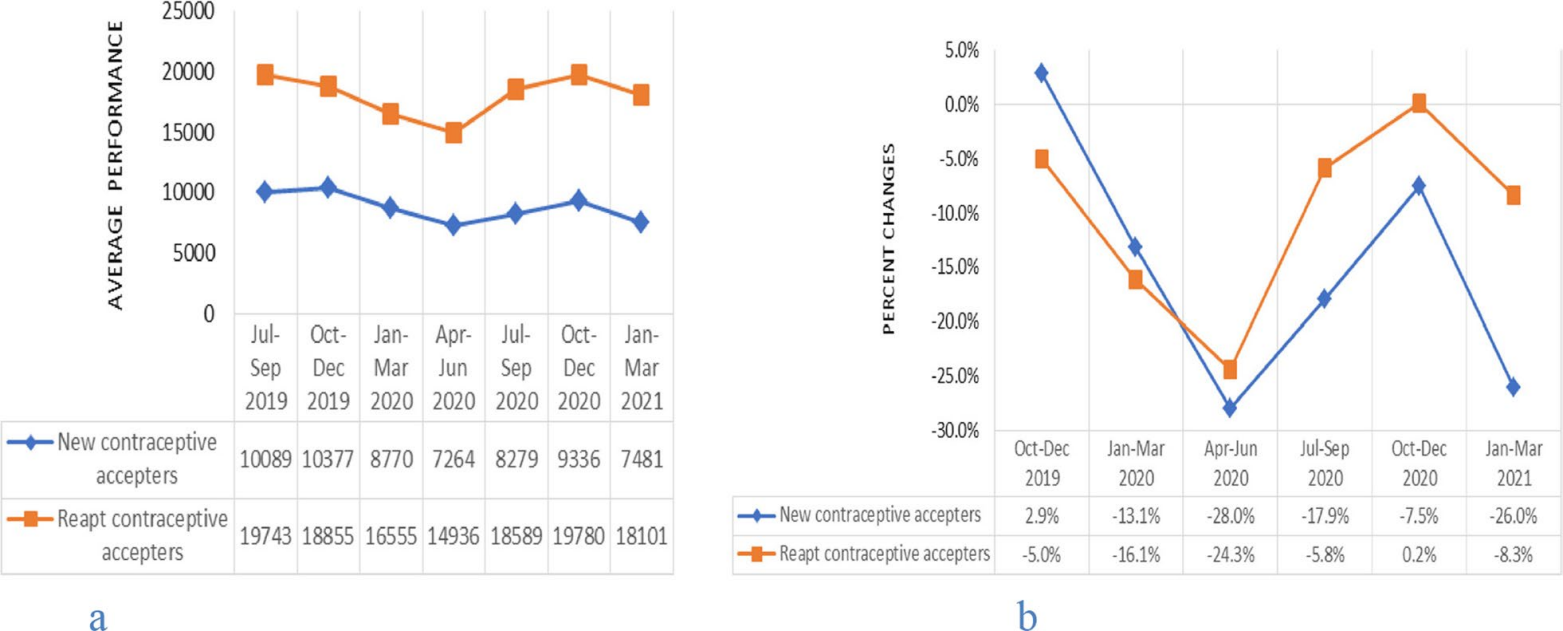
Trends in mean number and percentage changes in contraceptive accepters across quarters. **a** shows trends in mean number of contraceptive accepters across quarters from July 2019 to March 2021 and **b** shows percentage changes in contraceptive accepters across quarters from October 2019 to March 2021 compared to baseline (July–September 2019) average performance, Addis Ababa, Ethiopia

Trend analysis of contraceptives accepters showed that the average number of new contraceptives accepters began to decline during January–March 2020, with accelerated decline in the period April–June 2020. Repeat contraceptives accepters began to reduce during October–December 2019, and continuously decreased in the periods January to March 2020 and April to June 2020 (Fig. 2a). The percentage changes showed that new and repeat contraceptives accepters were reduced by 13.1%, 28%, 16.1% and 24.3%, respectively in the periods January to March 2020 and April to June 2020 compared to the July to September 2019 period average performance (Fig. 2b). During the periods July to September 2020 and October to December 2020, new contraceptives accepters continuously increased, but not to the pre-pandemic levels. The number of repeat contraceptives accepters began to increase during July–September 2020, with progressive increment between October–December 2020 to a level higher than the pre-pandemic level (Fig. 2a). In the last quarter of the study period (January–March 2021), new and repeat contraceptives accepters decreased by 26% and 8.3%, respectively compared to the July to September 2019 period (Fig. 2b). The percentage change in new and repeat contraceptives accepters for the period April–June 2020 was statistically significant with p-values 0.04 and 0.03, respectively.

**Fig. 3:**
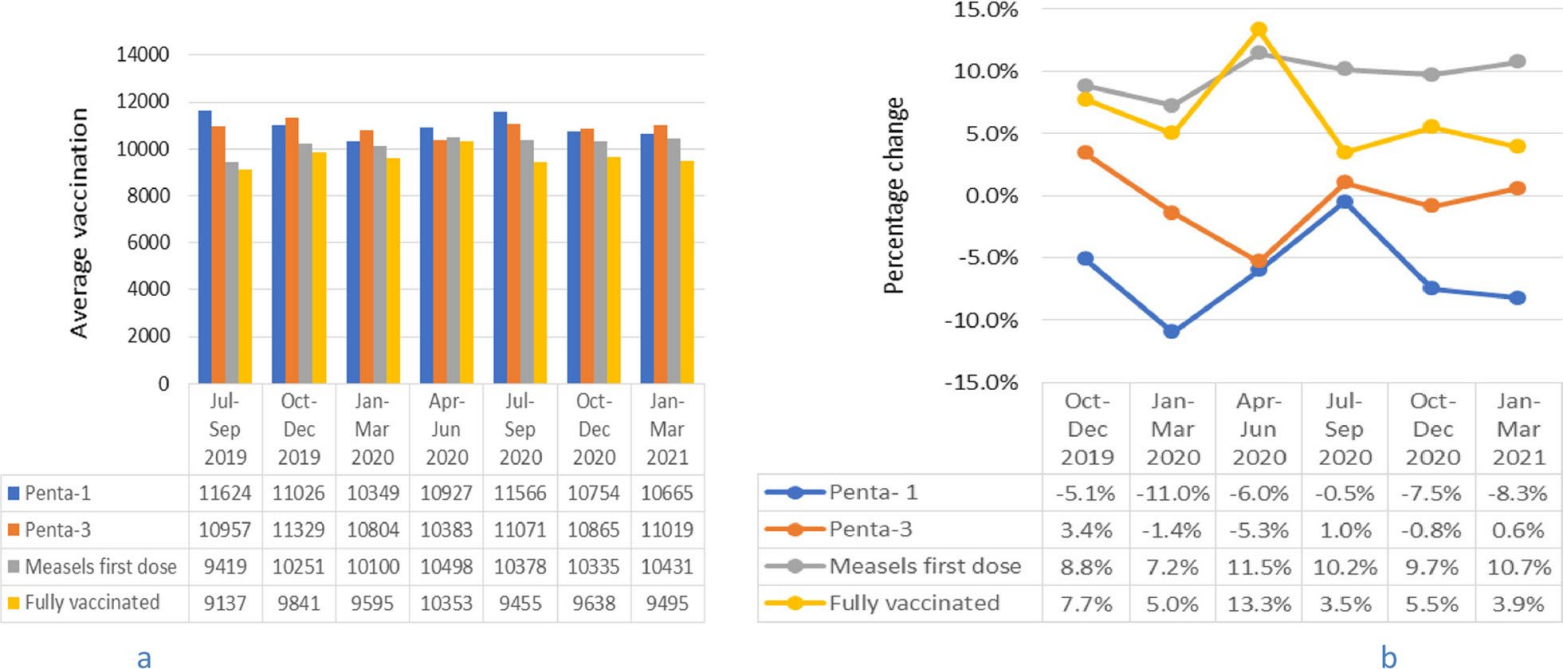
Trends in mean number and percentage changes in childhood vaccination across quarters. **a** shows trends in mean number of Pentavalent-1, Pentavalent-3, Measles first dose and full vaccination across quarters from July 2019 to March 2021 and **b** shows percentage changes in Pentavalent 1, Pentavalent-3, Measles first dose and full vaccination from October 2019 to March 2021 compared to baseline (July–September 2019) average performance, Addis Ababa, Ethiopia

Across quarters for the periods July 2019 to March 2021, Pentavalent-1 vaccination recipients decreased during October to December 2019, January to March 2020, October to December 2020 and January to March 2021. Pentavalent-3 vaccination recipients began to decrease during January to March 2020, with accelerated reduction in the period April to June 2020 (Fig. 3a). Percentage changes showed that Pentavalent-1 vaccination recipients declined by 5.1%, 11.0%, 7.5% and 8.3%, respectively in the periods October to December 2019, January to March 2020, October to December 2020 and January to March 2021 compared to the July to September 2019 period. Pentavalent-3 vaccination recipients decreased by 5.4% during April–June 2020 compared to the July–September 2019 period (Fig. 3b). The percentage change in Pentavalent-1 vaccination performance was statistically significant for the period October–December 2020 with p-value = 0.05. Pentavalent-1 vaccination performance recovered during April–June 2020 and July–September 2020 periods, but not to the pre-pandemic level. In the period July to September 2020, Pentavalent-3 vaccination performance recovered to a level higher than the pre-pandemic baseline then slightly declined during October–December 2020, but again increased in the period January–March 2021. Measles first dose and fully vaccinations remained higher than the pre-pandemic baseline levels across quarters in the periods October 2019 to March 2021. The highest increment was observed in April–June 2020 (Fig. 3a). Measles first dose and fully vaccinations increased by about 11.5% and 13.3%, respectively in the April–June 2020 (lock down) period compared to July–September 2019 (Fig. 3b).

**Fig. 4:**
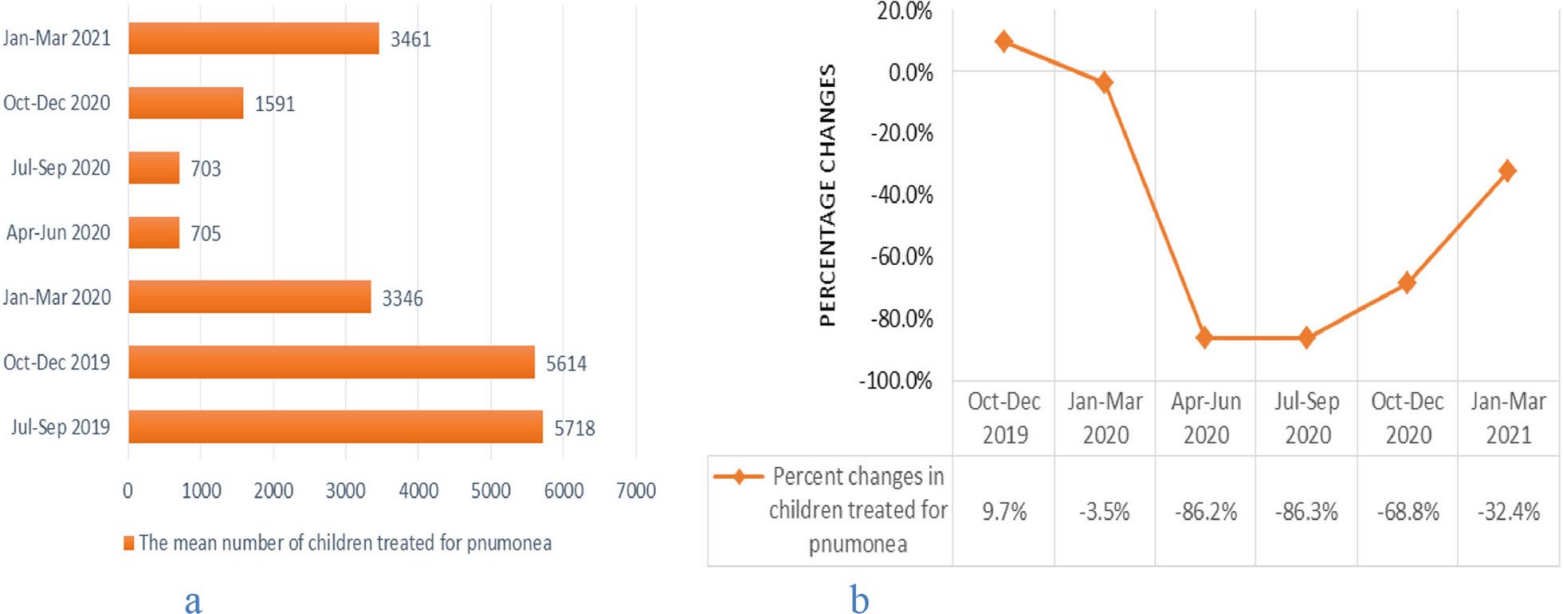
Trends in mean number and percentage changes in under 5 years old children treated for pneumonia across quarters. **a** shows trends in mean number of under-5 years old children treated for pneumonia across quarters from July 2019 to March 2021 and **b** shows percentage changes in the mean number of under-5 years old children treated for pneumonia from October 2019 to March 2021 compared to baseline (July–September 2019) average performance, Addis Ababa, Ethiopia

The average number of children under 5 years old treated for pneumonia began to decline in the period January to March 2020 with accelerated decline during April to June 2020 and July to September 2020 study periods (Fig. 4a). In the periods April to June 2020 and July to September 2020, the average number of children under-5 years old treated for pneumonia was reduced by 86.2% and 86.3%, respectively compared to the July to September 2019 period (Fig. 4b). The average number of children under-5 years old treated for pneumonia recovered during the period October–December 2020 and January–March 2021, but not to the pre-pandemic level (Fig. 4a). Compared to the baseline, the percentage change in average number of children under-5 years old treated for pneumonia was statistically significant with p-values 0.04, 0.01, 0.02 and 0.05 in the periods April–June 2020, July–September 2020, October–December 2020 and January–March 2021, respectively.

## Discussions

ANC first visit, health facility delivery, early PNC, new contraceptive accepters, repeat contraceptive accepters, safe abortion care, pentavalent-3 recipients and number of under-5 years old children treated for pneumonia decreased by 7%, 2.5%, 9.3%, 20.3%, 9.5%, 23.7%, 4.7% and 77.2%, respectively in the first 8 months of the COVID-19 pandemic compared to the pre-pandemic average performance. This might be related to inadequate supply of personal protective equipment, redirecting human workforce and services towards responding to COVID-19 pandemic. In addition, fear of acquiring COVID-19 by clients/patients if attended health facilities and financial barriers to seek health services might negatively affect services utilization [26, 27].

Early PNC visit significantly declined by 9.3% in the period March to October 2020 compared to the July 2019 to February 2020 period. Similarly, a study conducted in South West Ethiopia showed that PNC decreased by 29.1% in the period March–June 2020 [26]. PNC is a critical time for the well-being of mothers and newborns as most maternal deaths occur during the postpartum period, particularly within the first two days following delivery. Health professionals can use the opportunity to provide care while the mother is already in a health facility; however, this is the most neglected period for the provision of quality services [24, 28]. Likewise, safe abortion care significantly decreased by 23.7% in the same period. This finding is in line with a case study conducted at a tertiary facility in Addis Ababa whereby a decline of 14.5% was reported in safe abortion service during the period March–May 2020 [24]. On the other hand, in this study, post abortion care increased by 17.8% in the first 8 months during the COVID-19 pandemic. This is consistent with the results of a study conducted in another region of Ethiopia [27]. In Kenya 45% of pregnancies ended up with severe abortion complications during the COVID-19 pandemic [29]. The increased post abortion care in our study might be explained by women, who stayed at home during lockdown, were more prone to sexual abuse including rape even by their partners [30].

Institutional early neonatal death significantly increased by 13.3% in the period March–October 2020 compared to July 2019–February 2020. This is consistent with finding of a study done in Nepal [19]. In the Ethiopian context, institutional early neonatal death mainly defines the quality of obstetric care in a health facility [31]. The increase in institutional early neonatal death in this study may be related to compromised Intensive Care Units (ICUs) services; both space and equipment were redirected to COVID-19 care. Also, experienced ICUs staffs were more likely to be moved to COVID-19 treatment centers. Our study result underscores the importance of strengthening quality of health care services.

The percentage of Pentavalent-1 and Pentavalent-3 recipients and full vaccinated children decreased in the period March–October 2020 compared to the July 2019–February 2020 average performance. This is consistent with findings of study conducted in India [1]. The desire to decrease COVID-19 spread in health care facilities and the repurposing of health workers may have led to hesitation to continue routine immunization services in health facilities during the early period of the COVID-19 pandemic [20].

The trends in most of the services began to decline during January-March 2020, a quarter when COVID-19 began in Ethiopia. This was a time when the daily COVID-19 reported cases in Ethiopia, particularly in Addis Ababa were high [32]. In Ethiopia, as part of early response, there was intense media coverage about the COVID-19 outbreak, particularly in March 2020 after the country reported the first confirmed case. This might make the public fear of disease transmission and may have forced them to stop using health care services [27].

Some services including ANC four or more visits, early PNC visit, repeat contraceptive accepters and pentavalent-1 vaccination began to decline during October–December 2019 a quarter preceding the onset of COVID-19 pandemic in Ethiopia. The reductions in these services may be related to other reasons.

In this study, accelerated decline was observed in ANC first visit, ANC four or more visits, early PNC visit, new contraceptive accepters, repeat contraceptive accepters, pentavalent-3 recipients and the number of under-five children treated for pneumonia during April–June 2020 following national lockdown. The lockdown period in Ethiopia has been accompanied by measures including travel restrictions, flexible working arrangements, suspending public gathering and requirements for social distancing [10, 11]. This may have negative consequences on access to health services. Findings of studies in other regions of Ethiopia [11, 27] and other countries [17] also revealed decline trends of MCH services during lockdown.

ANC first visit declined during the first 8 months of the pandemic compared to the previous 8 months average performance. On the other hand, a relative increase was observed in ANC four or more visits during the same period. The possible explanation might be that the three previous ANC visits occurred before March 2020 when Ethiopia reported its first confirmed COVID-19 case. Trend assessment showed that both ANC first and four or more visits recovered and reached above the pre-pandemic baseline level average performance during the July–September 2020 (the last quarter of national lockdown) period. This may be related to an increased awareness of pregnant women about the disease transmission and its prevention method, which might have helped them to decrease their fear on COVID-19 and to visit health care facilities [11]. Also, a continuous services performance improvement across quarters in early PNC visit was observed since July–September 2020. In particular, in the last quarter of the study period (January–March 2021), recovery almost approached to pre-pandemic baseline level, and this was found to be an encouraging trend. These increasing trends in service provision may be due to efforts of the government to disseminate information on basic infection prevention control methods and guidance on safe care-seeking behavior [33]. Similarly, a study conducted in some districts of four regions in Ethiopia showed that most maternal health care services had already recovered by the end of July 2020 [11].

New contraceptive accepters significantly decreased by 20.3% and repeat contraceptive accepters decreased by 9.5% in the first 8 months of the COVID-19 pandemic. A study conducted in India showed a reduction in FP services in March 2020 compared to December 2019 [34]. Services performance recovery in contraceptive accepters was observed in the periods July–September 2020 and October–December 2020; however, new and repeat contraceptive accepters again began to decline and no recovery was observed in the period January–March 2021. Evidence indicates that a decrease in access to FP services results in increased poor outcomes related to unintended pregnancies and abortions [35]. The magnitude of unintended pregnancy during the COVID-19 pandemic among women attending ANC was 47.2% in Northwest Ethiopia [32].

The trend in Measles first dose vaccination across quarters remained above the pre-pandemic baseline level, although a slight positive decrement trend was observed during the periods January–March 2020, July–September 2020 and October–December 2020. Accelerated increment was observed in the period April–June 2020 following lockdown. This may be explained by the nationwide home to home campaign that was conducted in June 2020 in Ethiopia. About 15 million children have been vaccinated against measles in Ethiopia in an effort by the Ministry of health to maintain essential services despite COVID-19 challenges [36]. The country undertook the campaign taking into account the local COVID-19 transmission and implementing COVID-19 prevention measures [20]. Similarly, the trend in fully vaccination across quarters remained above the pre-pandemic baseline level with accelerated increment during April–June 2020 following lockdown. Nonetheless, positive decrement trend was observed in the periods July–September 2020 and at the end of the study period (January–March 2021).

Although increasing trend was observed in pentavalent-1 vaccination in April–June 2020 and July–September 2020, it continuously declined in the periods October to December 2020 and January to March 2021. A review of routine immunization data from 15 African countries showed that the number of children vaccinated with DPT1, DPT3 and MCV1 declined in the early period of the COVID-19 pandemic [20]. During an epidemic, even a temporary interruption of routine immunization services may lead to secondary health crises such as outbreaks during or after the recovery phase [37]. The deaths prevented by sustaining routine childhood immunization in Africa outweigh the excess risk of COVID-19 deaths associated with vaccination clinic visits, especially for the vaccinated children [38].

There has been a dramatic reduction in the average number of children under-5 years old treated for pneumonia in the first 8 months of the COVID-19 pandemic compared to the preceding 8 months. Also, we observed significant and accelerated decline trend in the periods April–June 2020 and July–September 2020 (during national lockdown). This may be due to the reduced under-five clinic services provision by health care facilities or reduced demand for health care services by clients/patients. However, in the periods October–December 2020 and January to March 2021 (after lockdown), slow reversal in a negative trend was observed, but does not appear to have recovered to the pre-pandemic baseline level. Pneumonia is the leading cause of under-five morbidity and mortality in Ethiopia, accounting 18.0% of all causes of mortality and killing over 40,000 children in under-five age group every year in the country [39]. Therefore, further study is warranted to investigate the reasons for the declining trend of under-5 years old children treated for pneumonia.

This study attempted to understand the performance status of selected RMNCH services using routine data in the context of COVID-19 pandemic. However, other studies showed high rates of inaccuracies in health records in Ethiopia [40, 41], and as we used routine HMIS data this study may share the inherent limitation of secondary data such as: inconsistency, incompleteness and inaccuracy. Nevertheless, extensive efforts have been made by the research team to ensure the data quality. The data collectors and supervisors cross checked the HMIS data at health facilities level whenever discrepancies occurred during the data review period.

## Conclusions

Most of the MCH services performance declined following onset of COVID-19 and national lockdown period in Addis Ababa, Ethiopia. Most of the MCH services began recovering in the period July–September 2020, the last quarter of national lockdown. In the last quarter of the study period, ANC first and four visits, PNC visit and the percentage of Pentavalent-3 recipients recovered to a level higher than the pre-pandemic baseline level. Measles first dose recipients and number of fully vaccinated remained above the pre-pandemic level across quarters. Although, new and repeat contraceptive accepters and pentavalent-1 recipients began recovering in July–September 2020, these services performance continued to decline and showed no recovery by end of the study period. The mean number of under-five children treated for pneumonia significantly declined across quarters, although slight recovery was observed in the last two quarters of the study period. This implies longer-term negative effects of COVID-19 on reproductive, maternal and child health services. Therefore, the Addis Ababa Health Bureau authorities should ensure the continuity of these services by implementing the required COVID-19 prevention measures and assuring the community about the safety of service delivery. Regular monitoring and evaluation of services performance is required to identify slowly recovering services and respond to potentially volatile changes during the COVID-19 pandemic.

## Data Availability

The datasets used and/or analyzed during the current study are available from the corresponding author on reasonable request.

## Abbreviations

AACAHB: Addis Ababa City Administration Health Bureau
AHRI: Armauer Hansen Research Institute
ANC: Antenatal care
COVID-19: Coronavirus disease 2019
FP: Family planning
HMIS: Health management information system
MCH: Maternal and child health
RMNCH: Reproductive, maternal, neonatal and child health
PNC: Postnatal care
SARS-CoV-2: Severe acute respiratory syndrome coronavirus 2
SBA: Skilled birth attendance
WHO: World Health Organization
